# The Burden of Zoonoses in Kyrgyzstan: A Systematic Review

**DOI:** 10.1371/journal.pntd.0004831

**Published:** 2016-07-07

**Authors:** Michel J. Counotte, Gulnara Minbaeva, Jumagul Usubalieva, Kubanychbek Abdykerimov, Paul R. Torgerson

**Affiliations:** 1 Section of Epidemiology, Vetsuisse Faculty, University of Zurich, Zurich, Switzerland; 2 Faculty of Veterinary Medicine, Utrecht University, Utrecht, The Netherlands; 3 State Sanitary Epidemiological Department of the Kyrgyz Republic, Bishkek, Kyrgyzstan; 4 Laboratory "Express Plus", Bishkek, Kyrgyzstan; McGill University, CANADA

## Abstract

**Introduction:**

Zoonotic disease (ZD) pose a serious threat to human health in low-income countries. In these countries the human burden of disease is often underestimated due to insufficient monitoring because of insufficient funding. Quantification of the impact of zoonoses helps in prioritizing healthcare needs. Kyrgyzstan is a poor, mountainous country with 48% of the population employed in agriculture and one third of the population living below the poverty line.

**Methodology/Principal Findings:**

We have assessed the burden of zoonoses in Kyrgyzstan by conducting a systematic review. We have used the collected data to estimate the burden of ZDs and addressed the underestimation in officially reported disease incidence. The estimated incidences of the ZDs were used to calculate incidence-based Disability Adjusted Life Years (DALYs). This standardized health gap measure enhances comparability between injuries and diseases. The combined burden for alveolar echinococcosis, cystic echinococcosis, brucellosis, campylobacteriosis, congenital toxoplasmosis, non-typhoidal salmonellosis and rabies in Kyrgyzstan in 2013 was 35,209 DALYs [95% Uncertainty interval (UI):13,413–83,777]; 576 deaths [95% UI: 279–1,168] were attributed to these infections. We estimate a combined median incidence of ZDs of 141,583 cases [95% UI: 33,912–250,924] in 2013. The highest burden was caused by non-typhoidal *Salmonella* and *Echinococcus multilocularis*, respectively 14,792 DALYs [95% UI: 3,966–41,532] and 11,915 DALYs [95% UI: 4,705–27,114] per year.

**Conclusion/Significance:**

The health impact of zoonoses in Kyrgyzstan is substantial, comparable to that of HIV. Community-based surveillance studies and hospital-based registration of all occurrences of zoonoses would increase the accuracy of the estimates.

## Author Summary

Zoonoses are diseases transmitted from vertebrate animals to humans. They can cause a variety of symptoms ranging from mild gastrointestinal complaints to debilitating illness and even death. Especially in low-income countries where animals play an important role for many, the burden of these diseases can be substantial. However, there is often little attention for these diseases, thus they remain under-researched and underfunded. In this review, we present estimates of the burden of the most important zoonotic diseases in Kyrgyzstan for the reference year 2013. We estimated the burden by calculating the incidence-based disability adjusted life years (DALYs), allowing comparison between diseases and injuries. Disease frequency data is scarce and hospital-based incidence data often underestimates the true incidence of the disease. By addressing the underestimation in officially reported incidence using data from our systematic review, we estimated the true incidence of the most important zoonoses in Kyrgyzstan. We quantified the substantial impact these diseases have on the wellbeing of people in Kyrgyzstan in 2013. The results underline the need for more intensive monitoring and surveillance of zoonotic diseases.

## Introduction

Zoonoses are diseases in humans, which are naturally transmissible directly or indirectly from vertebrate animals. Of 1415 species of infectious organisms know to be human pathogens, 61% are zoonotic [1]. The Food and Agriculture Organization of the United Nations (FAO), the World Health Organization (WHO) and the World Organisation for Animal Health (OIE) have underlined the important socioeconomic impact of these diseases, yet in many low income countries the burden of zoonoses remains unknown [2]. The lack of information often results in a vicious circle of underestimation and limited incentive to quantify the problem [3,4].

**Fig 1:**
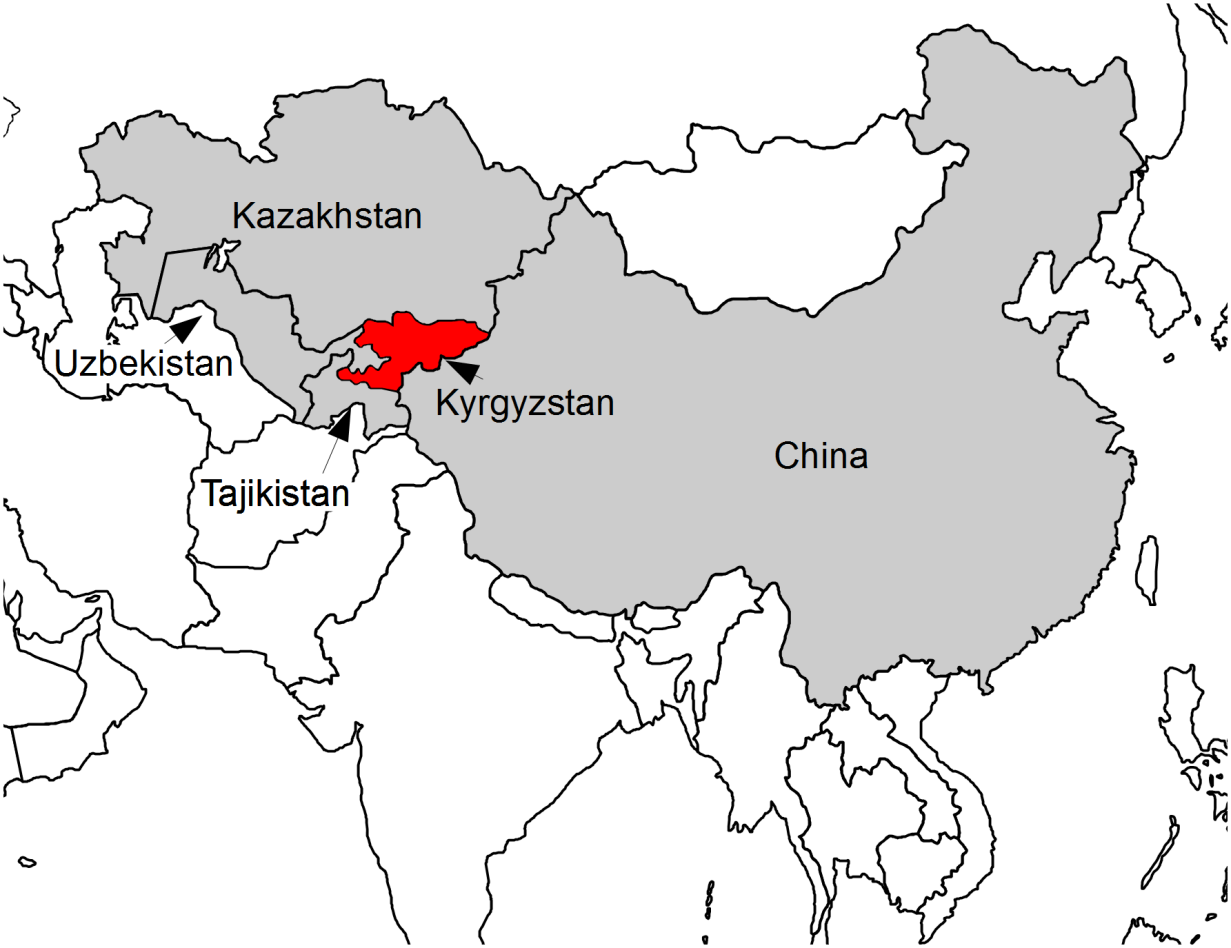
Kyrgyzstan (in red) is bordered by China in the East, Kazakhstan in the north, Uzbekistan and Tajikistan in the south-west (grey).

Kyrgyzstan is a country in Central Asia, neighboured by China in the west, Kazakhstan in the north, and Tajikistan and Uzbekistan in the southeast (Fig 1). Because of a poor functioning veterinary and sanitation system, emerging zoonoses are an increasing problem [5]. Since independence in 1991, veterinary services deteriorated, causing an increase in zoonotic disease (ZD) [6,7]. At particular risk are the 64% of the inhabitants who live in rural areas, where livestock farming plays an important role. Seventy-six percent of these rural dwellers are considered to be poor [8]. The small-scale farming, which is often nomadic, allows intensive contact between humans and animals [9].

Furthermore, in Kyrgyzstan, an estimated 100 DALYs per 100,000 were lost due to inadequate hygiene in 2012 [10], which ranks the country in the 61th place out of 146 low/middle-income countries on disease burden due to poor hygiene.

The combination of poor healthcare, poverty, inadequate hygiene, and the close interaction between humans, livestock and other animals, leaves a large share of the population at risk of being infected with zoonoses.

Another difficulty in assessing the burden of the diseases, is the low scientific output from Kyrgyzstan which is often published in Russian [11,12].

The World Bank and the OIE have advised Kyrgyzstan to develop national animal disease control strategies [2]. A quantification of the impact of zoonoses helps prioritizing these diseases. The aim of this study was to quantify the burden of ZD in Kyrgyzstan using disability-adjusted life years (DALYs) a standardized approach to increase comparability of disease impact [13–19].

In this review, we have assessed the available data on zoonoses in Kyrgyzstan with special attention to the potential underreporting using stochastic disease modelling. We have comprehensively summarised the burden of the most important zoonoses that are endemic in Kyrgyzstan and addressed the underestimation in officially reported cases.

## Materials and Methods

### Selection of zoonotic diseases

The ZDs described in this systematic review (Table 1) are regarded as the most important in terms of socioeconomic impact based on the WHO report on neglected tropical diseases, the World Bank report on Kyrgyzstan of 2011 and other systematic reviews of neighbouring or overlapping regions [2,5,20,21].

**Table 1:** List of important zoonoses in Kyrgyzstan.

Zoonoses	Causative agent	Main animal source	ref
Anthrax	*Bacillus anthracis*	Cattle, sheep, goats	[22]
Brucellosis	*Brucella* spp.	Cattle, sheep, goats	[23,24]
Alveolar echinococcosis	*Echinococcus multilocularis*	Dogs, (foxes)	[25,26]
Cystic echinococcosis	*Echinococcus granulosus*	Dogs	[25,26]
Rabies	Rabies virus	Dogs, (other mammals)	[27]
Toxoplasmosis	*Toxoplasma gondii*	Sheep, goats, cats	[28]
Non-typhoidal salmonellosis	*Salmonella* spp. (except for serotypes Typhi and Paratyphi.)	Poultry, cattle	[29]
Campylobacteriosis	*Campylobacter* spp.	Poultry, cattle, sheep	[30]

### Systematic review

We assembled all the available evidence regarding prevalence or incidence of the selected ZDs in Kyrgyzstan since the country became independent. Therefore, the time period for the search was January 1991-January 2016. Both formal, peer-reviewed scientific literature, and informal sources, grey literature, were considered. A full list of sources can be found in S1 Supporting Information. We conducted a systematic review by following guidelines of the Preferred Reporting Items for Systematic reviews and Meta-Analyses (PRISMA guidelines, Moher, Liberati, Tetzlaff, Altman, & The PRISMA Group, 2009) [31] (S2 Supporting Information– Prisma checklist).

A list of synonyms for the ZDs was constructed using the pathogen’s name and alternative (popular) names of the disease. The computer search was constructed by combining these terms with the Boolean OR and the term ‘Kyrgyzstan’ with the AND Boolean. The databases of PubMed, Google Scholar, Web of Science, OVID, Scopus, the WHO Global Health Library, Food and Agriculture Organization of the United Nations (FAO) and ProMED-mail were searched using English search terms and Google Scholar using Russian search terms. For each database, the search construct was adapted to the specific modus operandi of the search engine. S1 Supporting Information lists the search terms as well as the search constructs for the different databases.

Furthermore, we searched the internet for published reports on demographic surveillance sites in the English and the Russian language. Data from government sources was contributed by the co-authors.

**Fig 2:**
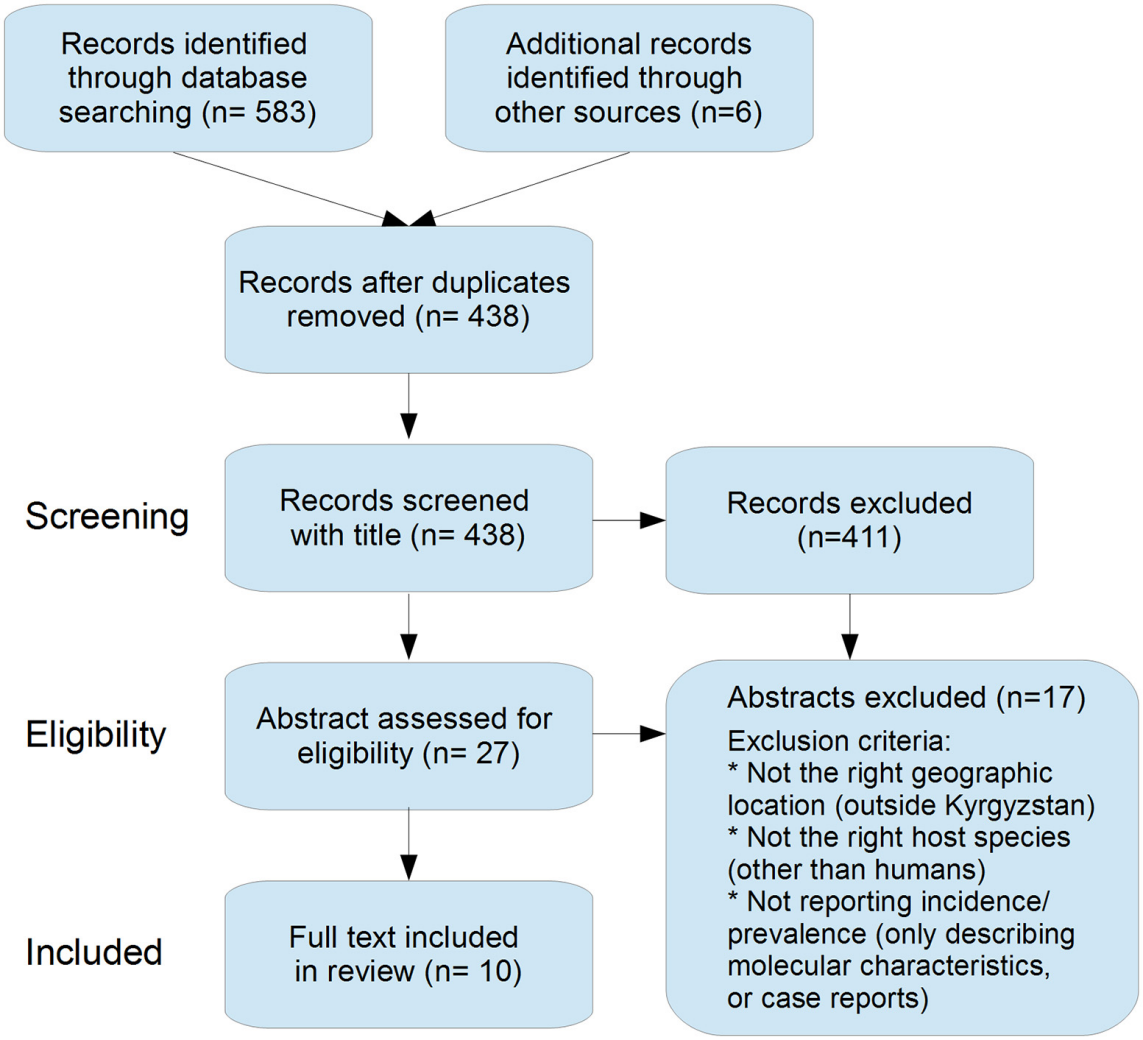
Flow diagram of the results of the systematic search of the literature for the burden of zoonotic diseases in Kyrgyzstan, adapted from [31].

Following retrieval, studies were selected by critically appraising the titles and abstracts. A study was excluded when it did not address prevalence or incidence for the specific disease, when it was not from the defined period, when it did not address the disease in humans or when it did not address Kyrgyzstan. Secondly, the full text was screened and for each retrieved result the list of references was inspected for additional sources (backward searching). Forward searching was performed by entering the titles in google scholar using the ‘cited by’ function. Searches were executed until no new results were found. Additional results were screened according to the same methodology. Finally, the selected studies were summarized based on study design, study area, disease measure (prevalence/incidence) and the reported margin of error (S3 Supporting Information). Each study was critically appraised on methodology, selectivity in reporting and assumptions made by the authors. Fig 2 displays a flow diagram of the used selection strategy.

### DALY calculation—burden assessment

The Disability-Adjusted Life Year (DALY) was used as the burden-of-disease metric. It is a health gap measure which quantifies health loss. DALY calculation is a standardized method developed by the World Bank, Harvard School of Public Health and the World Health Organization for the Global Burden of Disease and injury (GBD) study and the global burden of foodborne diseases [13,32–35]. It allows the comparison of health conditions across countries and across diseases [13,32]. In this study, an incidence-based DALY calculation was applied. This allows us to include all sequelae resulting from infection [36,37].

The DALYs are calculated as the sum of the healthy years lost to disability (YLD) and the years of life lost due to premature death (YLL). The YLD is the sum of the different outcomes that result in disability, where an outcome is defined as sequelae of the disease or another categorisation of the disease, e.g. chronic vs. acute. The YLD per outcome is the product of the duration, the incidence, and the disability weight of the outcome. The YLL is the residual life expectancy at the age of death.

YLLs were calculated based on the life table from [38]. YLDs with a lifelong duration were calculated based on a local life table from the WHO for Kyrgyzstan for 2013 [39]. Based on the recommendations and methodology of the GBD 2010 and FERG [33,36], we have used a non-discounted and non-weighted approach in calculating DALYs.

If a disease and its outcomes were quantifiable, a corresponding disease outcome model was constructed based on literature. When data on the incidence, the outcome of a disease, or other parameters for the DALY calculation were missing, these gaps were filled using data from neighbouring countries or overlapping regions.

The disease model or outcome-tree model was constructed per ZD using health outcomes with an evidence-based causal relationship between infection and outcome. Disagreement in inputs of the disease model between different sources was modelled using distributions (pert, triangular, and uniform) accounting for this uncertainty. A full description of the disease models, the input parameters and its uncertainties used to calculate the DALYs can be found in S3 and S4 Supporting Information.

Where available, disability weights from the GBD 2010 study were used. Age distributions of outcomes were, when possible, based on data collected from Kyrgyzstan. Furthermore, the total population size, age and sex distribution was obtained from census data by the National Statistical Committee of the Kyrgyz Republic and the United Nations Demographic Yearbook [40,41].

The uncertainty in the estimates was modelled using Monte Carlo analysis. We generated in this simulation 10,000 draws from the probability distributions. All analyses were performed using R version 3.2.2 (R Foundation for Statistical Computing, Vienna, Austria) [42]. Additional information on the analyses and disease models is provided in S4 Supporting Information. The burden of disease was calculated for the reference year 2013, a result of a trade-off between data availability and as recent as possible.

The data collected by the Department of State Sanitary and Epidemiological Supervision of the Ministry of Health of the Kyrgyz Republic provided the officially reported cases for notifiable diseases which includes a number of zoonoses. This is available online [41,43]. We have used the data retrieved from literature to evaluate these reported figures and assess the level of underestimation. As reported in S3 Supporting Information, the availability of published disease data is scarce in Kyrgyzstan; we were not able to assess the burden of anthrax since not enough was known about the outcome of the cases. In 2013, 16 human cases of anthrax were reported. The majority of the cases were the cutaneous form, Zoldoshev reviewed 217 cases of cutaneous anthrax with no fatalities [44]. For alveolar echinococcosis (AE), cystic echinococcosis (CE), brucellosis and rabies incidence data for Kyrgyzstan was available from the Department of State Sanitary and Epidemiological Supervision of the Ministry of Health of the Kyrgyz Republic [41,43]. Prevalence data on AE, CE and brucellosis was used to address the underestimation in the official data; to address the potential underestimation in rabies we have used data from overlapping regions (Eurasia) [27]. The incidence of congenital toxoplasmosis was not formally recorded, but Minbaeva et al. (2013) provided estimates for Kyrgyzstan [45].

For campylobacteriosis and non-typhoidal salmonellosis, no specific disease prevalence or incidence data was recorded for Kyrgyzstan. The incidence estimates are based on the number of acute gastrointestinal infections reported in 2013 [41] and the assumed etiological fraction as described by [21, 22]. This conservative estimate was used as the lower limit for the number of cases; the upper limit was formed by the etiologic proportion of the diarrhoea incidence multiplied by the gastroenteritis incidence from the European region based on Walker et al. and Lanata et al. [24–27]. Invasive non-typhoidal salmonellosis (iNTS) forms an important outcome of non-typhoidal salmonellosis infection since mortality is much higher compared to the gastro-enteric manifestation of the disease [35,46]. However limited data is available on the true incidence of iNTS since only few population-based incidence studies have been conducted. Therefore, we have used the ratio between iNTS:NTS as described by Ao et al. [46] who classified Kyrgyzstan in the Asia/Oceania region where the proportion iNTS:NTS was 1:3,851 compared to 1:7 in European region which included Russia; the global average ratio was 1:28 [46].

## Results

### Systematic review

We identified 438 unique citations and excluded 411 by title and abstract screening. Of the remaining 28 potential eligible citations with relevant abstracts, 10 were eligible for full text review. The PRISMA flowchart summarizing the data collection process is presented in Fig 2. Reports published during January 1991-January 2016 were searched. The last search was performed on 19-02-2016. All collected data are summarised in S3 Supporting Information.

### Burden assessment

**Fig 3:**
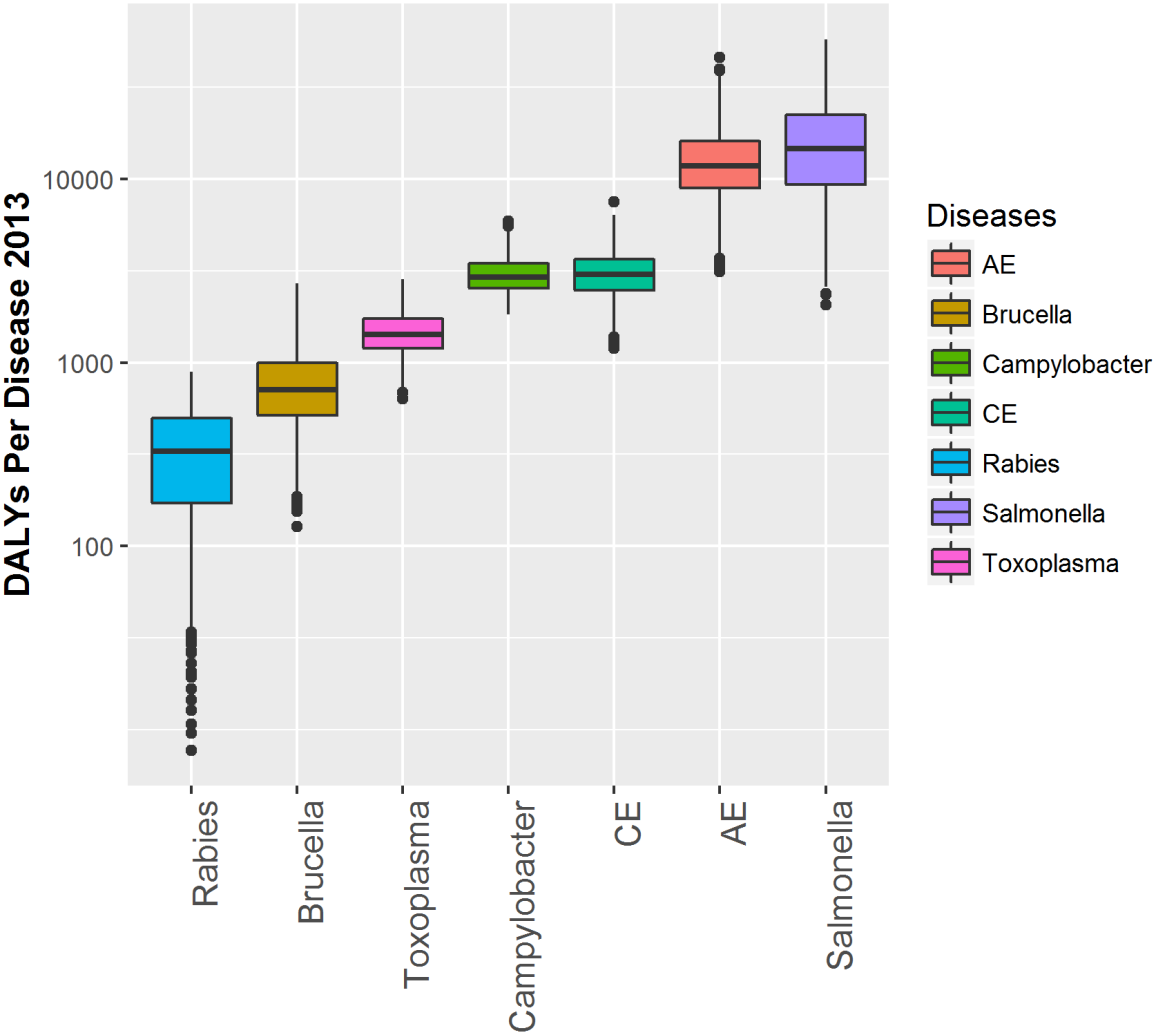
The estimated impact in DALYs per year for 2013 in Kyrgyzstan for seven zoonotic diseases. Ranking of ZDs by yearly burden: Rabies, Brucellosis (Brucella), Congenital toxoplasmosis (Toxoplasma), Cystic echinococcosis (CE), Campylobacteriosis (Campylobacter), Alveolar echinococcosis (AE), non-typhoidal Salmonellosis (Salmonella). The boxplots show the median, quartiles, and outliers per ZD.

**Fig 4:**
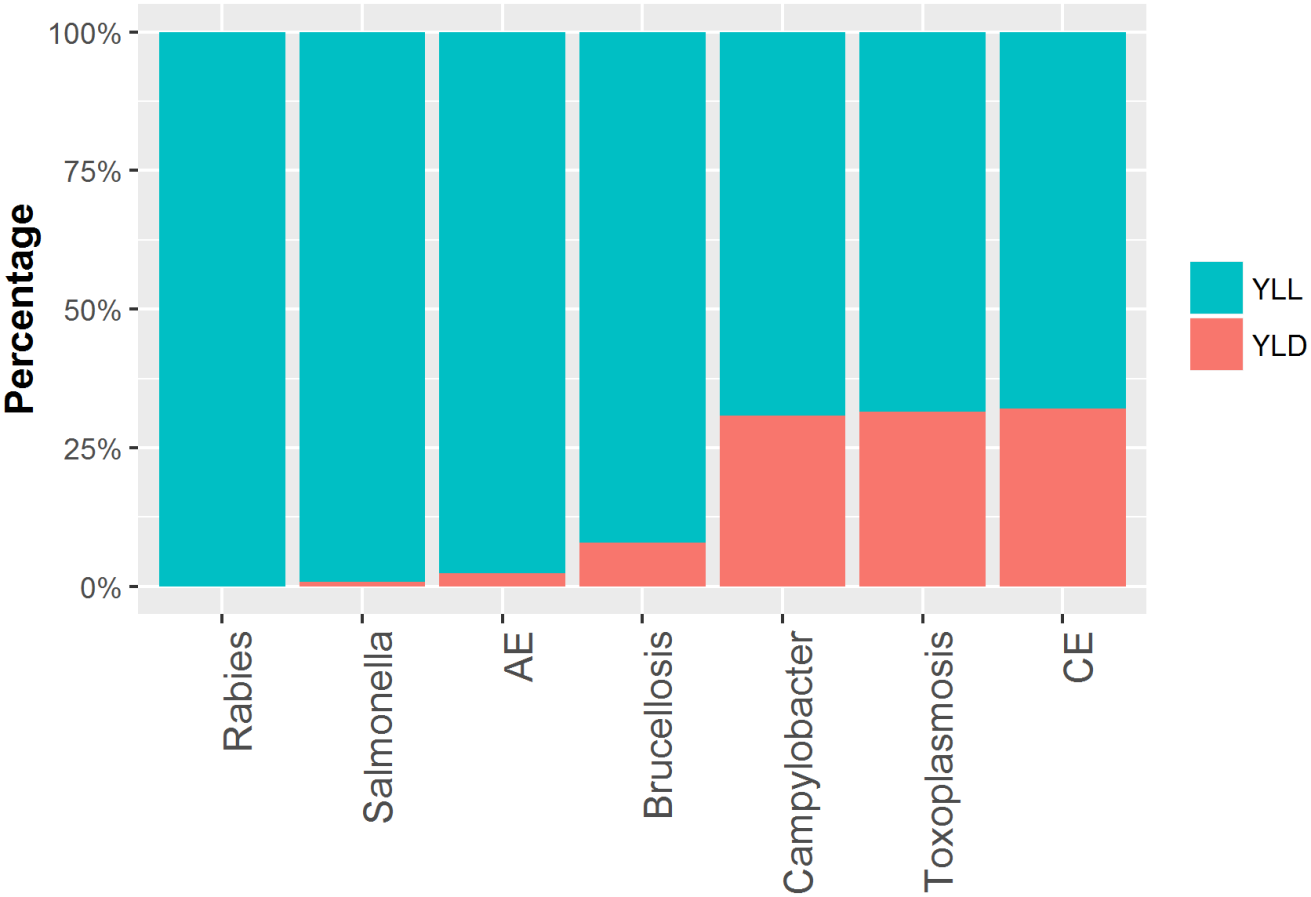
The proportion YLD and YLL per disease for seven zoonotic diseases quantified in Kyrgyzstan for 2013.

In 2013 seven ZDs were quantifiable in Kyrgyzstan. AE, brucellosis, campylobacteriosis, CE, congenital toxoplasmosis, NTS and rabies. These were responsible for an estimated total of 141,583 [33,912–250,924] new cases resulting in 35,209 [13,413–83,777] DALYs and 576 [279–1,168] deaths (Table 2, Fig 3). Both Rabies and AE contribute a large number of DALYs per case, 70.1 [10.0–90.0] DALYs/case and 50.3 [20.7–78.3] DALYs/case respectively, due to high mortality (Fig 4). Campylobacteriosis and NTS had relatively low mortality but a high incidence; most of the mortality was due to the sequelae Guillain Barre Syndrome (GBS) and invasive non-typhoidal salmonellosis (iNTS) respectively, see S4 Supporting Information. Infections with salmonellosis and AE were responsible for the majority of deaths, respectively 254 [66–571] and 236 [153–466]. Although only 5.1% (11/216) of the cases of congenital toxoplasmosis was fatal, the DALY/case is high (6.69) due to early onset of the sequelae and the lifelong duration. Table 2 provides the estimates for the number of cases, the DALY, the number of deaths and the DALY per case per disease and their 95% uncertainty range. Fig 3 displays a graphical representation of the per annum burden per disease and its uncertainty, plotting the DALY estimates from Table 2 per disease. Table 3 displays the sensitivity analysis with different iNTS:NTS ratios. Fig 4 provides the percentage of YLD and YLL per disease. Premature mortality, or YLL, (in blue in Fig 4) contributes for all diseases most to the DALY, ranging from 67% for CE to 100% for Rabies.

**Table 2:** Estimated median incidence (cases), median DALY per year for 2013 and median deaths attributed to infections in 2013 per disease in Kyrgyzstan with corresponding 95% uncertainty intervals.

Disease	Cases	95% range	DALY	95% range	Deaths	95% range	DALY/case	95% range
Campylobacteriosis	73,953	17,344–130,705	2,956	2,057–5,068	24	21–28	0.04	0.023–0.13
AE	236	153–466	11,915	4,705–27,114	236*	153–466	50.3	20.7–78.3
CE	2,382	1,876–3,760	3,052	1,508–5,205	33	28–47	1.25	0.62–1.69
Salmonellosis	62,355	12,892–111,273	14,792	3,966–41,532	254	66–571	0.27	0.12–0.55
Brucellosis	2,435	1,467–4,449	713	257–1,747	12	5–26	0.29	0.12–0.54
Toxoplasmosis	216	179–261	1,448	874–2,320	11	5–20	6.69	4.17–10.23
Rabies	6	1–10	333	46–791	6	1–10	70.1	10.0–90.0
Total:	141,583	33,912–250,924	35,209	13,413–83,777	576	279–1,168	---	--

* Assumed to be fatal, although there may be a period of several years between diagnosis and death.

**Table 3:** Sensitivity analysis with different ratios of invasive non-typhoidal salmonellosis (iNTS): non-typhoidal salmonellosis (NTS). Estimated median DALYs, DALY/case and median cases per year in 2013 in Kyrgyzstan for non-typhoidal salmonellosis based on different proportions iNTS:NTS based on Ao et al. [46].

iNTS:NTS	DALY median	DALY/case	Cases*
1:28	13,348	0.2429	61939
1:7	48,226	0.783	67775
1:3,851	1,744	0.0294	59954

* Cases are defined as the sum of the estimated per annum incidence of both (enteric) NTS and iNTS

Detailed description of the parameter estimates, their distributions and the calculation of the disease incidence as used in the DALY calculations can be found in S4 Supporting Information.

## Discussion

This work provides a first attempt at quantifying the burden of ZD in Kyrgyzstan. It underlines the lack of published data on many zoonoses in this region. However, the estimates of the impact of the ZDs help to break the vicious circle of underreporting by providing estimates of the true incidence and burden of these diseases.

Because of the scarcity of data we did not exclude information based on methodology; we analysed it using conservative assumptions and stochastic modelling to handle uncertainty [47]. We have used official Kyrgyz data and addressed its underestimation.

The total burden of the seven quantified ZDs (35,209 [13,413–83,777] DALYs in 2013) is slightly less than the yearly burden of HIV, which was attributable for 38,870 [21,261–64,297] DALYs in 2010 in Kyrgyzstan [48]. This burden is based on prevalence based DALYs, as used in the GBD 2010 studies [33].

Forty-three percent of the estimated burden of zoonoses or 14,967 [6,213–32,319] DALYs in 2013, in Kyrgyzstan is caused by echinococcosis (both AE and CE). Torgerson et al. estimated in 2010 that in China 16,629 new cases of AE per year arose among 22.6 million people at risk [17]. In Kyrgyzstan we estimate for 2013 that 236 cases arose among 5.7 million people. However AE is characterized by a clustered distribution and some regions have a much higher incidence rate [49]. The officially reported incidence of AE has increased since 2004 at an alarming rate. Where before 2004 only 0–3 cases per year were reported, in 2013 148 cases were officially reported [49,50]. We assume that, corrected for underestimation, the incidence is likely to be approximately 236 [153–466] cases in 2013.

Although the goal of this study was to provide an estimate of the burden of zoonoses in 2013, the collected data allows us to reflect on temporal trends. Raimkylov & Kuttubaev, and Usubalieva et al. describe an increasing trend over the last decade in the incidence of echinococcosis [49,50], although increased awareness might lead to more diagnoses. The yearly incidence of brucellosis, on the other hand, seems to decline over time (S3 Supporting Information). The application of the ocular Rev-1 vaccination over several years has most likely resulted in this decreased incidence [51]. Over time, the improvement of diagnostics and the application of novel treatments may cause changes in the outcome of the disease and thus the DALY per case. For example, in our analysis, we assumed that all cases of AE are eventually fatal due to insufficient treatment. However, as illustrated in Switzerland, adequate treatment of the disease will lead to an increased survival [52]. This illustrates the need for periodic updating of the burden assessment.

We choose an incidence-based approach in the DALY modelling because it allows us to include all sequelae resulting from infection. However, one of the consequences of using the incidence-based DALY approach, is that deaths in the future are attributed to the year of the infection. Careful interpretation of the mortality rate is therefore advised. For example, AE did not cause the reported number of deaths in 2013 since its incidence is increasing and it has a long latency; the deaths that will be caused by the infections diagnosed in 2013 are attributed to that year. For a disease with a short incubation time and a relative constant incidence rate, such as rabies, the difference is not so striking.

Using a prevalence based DALY approach in diseases with a trend over time and a long latent phase or incubation period, might lead to under or overestimation as it reflects past infection rather than a present day event [37].

Another limitation is the assumption, in common with other burden studies, that the outcome of diseases can be extrapolated to different countries. Regional differences in pathogens might change the tropism of the causative agent or cause a shift towards certain sequelae. The same holds true for other spatially fluctuating factors, such as co-infection; The incidence of iNTS, for example, is correlated with malaria and HIV infection [46]. This underlines the importance of not only reporting incident cases, but also of documenting disease outcome. Other factors such as ethnicity might also have an influence on disease outcome, as for example has been postulated in tuberculosis and *Plasmodium falciparum* malaria [53,54]. Even more striking are the vast differences in treatment according to region, as illustrated for AE. Likewise, brucellosis, with inadequate treatment is more likely to become chronic or relapse [55] which increases the burden.

Underestimation of disease is caused by under-ascertainment and underreporting of cases. A disease might not be severe enough for the patient to visit a medical facility. In addition, patients might have limited access to care or the disease are not accurately diagnosed. Underreporting is the result of incomplete registration of cases. Even in countries with a high standard of medical care, such as WHO high-income countries, reported cases form only the tip of the iceberg of the true incidence. For example, it is estimated that only 1/30.3-1/86 cases of campylobacteriosis are reported in the USA [56,57]; the estimate in the European Union is that on average 1/47 cases of campylobacteriosis are reported [58]. CE is often substantially underreported. In Uzbekistan the official case numbers appear reported were 1,435 cases reported in 2000 and 819 cases reported in 2001 [59]. However, Nazirov and others undertook a detailed study of hospital records throughout Uzbekistan and found a total of 4,430 cases in 2000 and 4,089 cases in 2001. Likewise in Chile official notifications between 2001 and 2009 were a mean of 311 cases per annum, whilst a detailed audit of hospital records revealed a mean of 1,009 cases per annum [60].

The assumption we made on the underestimation of the incidence of ZDs is conservative. For most ZDs we have used either a uniform or a pert distribution and included the officially reported incidence as minimum and the with a multiplication factor corrected value as maximum. The assumption for the mode in AE, CE and brucellosis are also conservative. The multiplication factor we used to correct for underestimation in brucellosis (4.6) lies close to the mean multiplication factor (5.4 [1.6–15.4]) Kirk et al. used in [35].

Hampson et al. reviewed the global burden of Rabies and estimated for 2010 and estimated 14 rabies deaths in Kyrgyzstan contributing to 887 DALYs [27]. Since rabies is a fatal disease, which often affects the young, it is possible that some cases go unreported in Kyrgyzstan. We believe that our estimate and its 95% uncertainty range represent the true incidence. The burden consists only of the estimated fatal cases, and not the disability caused by dog bites and the burden of the treatment. This indirect burden is highest in countries where crude nerve-tissue vaccines are used [61], which is not the case in Kyrgyzstan. Other carnivores than dogs, are assumed not be relevant in contributing to the transmission risk [62], however, there is a steep increase described in the wolf population and an increasing contact rate between humans and these wild carnivores in mainly in the south of Kyrgyzstan [63].

In this study we have explored the proportion of diarrhoea attributable to *Campylobacter* and non-typhoidal *Salmonella* in Kyrgyzstan. We assumed etiologic proportion of diarrhoea of both pathogens based on literature [64]. Close inspection of the reported incidence of acute intestinal infections, reveals an approximate two-fold increase in cases between 2004 and 2007. However, the change was likely because the funding of hospitals was modified to a case-based system [65]. This illustrates that the variance in reported data does not always represent epidemiological change; it can be merely a reflection of an alteration in policy. The estimates we present are based on conservative extrapolates from overlapping regions. However, more accurate incidence data on salmonellosis and campylobacteriosis in Kyrgyzstan are lacking. Estimates of overlapping regions often lacked nuance and tend to group heterogeneous countries. Our median incidence estimates for both NTS (1,101/100,000) and campylobacteriosis (1,305/100,000) are higher than the estimated yearly incidence by Havelaar et al. of campylobacteriosis (802/100,000 cases) and of NTS (318/100,000 cases) in the EUR B region [66]. However earlier estimates by the same author are higher; ranging from 1,800–11,800 cases/100.000 for salmonellosis and 2,240–13,500 cases/100.000 for campylobacteriosis [58]. The data presented in [58] shows a correlation between Gross Domestic Product (GDP) and both salmonellosis and campylobacteriosis; both diseases have a higher estimated true incidence in countries with a lower GDP. There seems to be no clear relation between the quantity of consumed protein (egg, chicken, and pork) according to the FAO and the estimated true incidence of the two diseases in the different European countries (EU-27) [58]. We believe that although chicken, egg and pork consumption in Kyrgyzstan are lower than in EU-27 countries, the lower GDP and the lower hygiene standard in Kyrgyzstan justify our estimates.

To obtain more reliable burden estimates of both campylobacteriosis and salmonellosis, it would be advisable to undertake a community-based incidence study in Kyrgyzstan. Both diarrhoea incidence and aetiology are important inputs to narrow the uncertainty around our estimates. Furthermore, a longitudinal study on the aetiology of febrile illness might provide a reliable estimate of the burden of different zoonoses or sequelae (brucellosis, iNTS, listeriosis, Q-fever, leptospirosis). A small scale investigation in Bishkek showed that part of the undiagnosed febrile illness was due to brucellosis and Q-fever [67].

To date, the exact burden of leptospirosis in Kyrgyzstan is unknown. Although occurrence of leptospirosis in cattle in Kyrgyzstan has been reported [68], no data on the occurrence of this ZD in humans in Kyrgyzstan is available. Torgerson et al. estimated that the burden of leptospirosis in Kyrgyzstan was 927 [355–1629] DALYs per year [69]. Costa et al. clearly illustrate a lack of data on the occurrence of leptospirosis in Central Asia; the estimates of incidence for Kyrgyzstan were based on extrapolation using a multivariable regression model [70].

Likewise, the role of cryptosporidium and giardia as causative agent for ZD in Kyrgyzstan has not been established. These parasites have zoonotic potential [71], however the incidence of the disease caused by these parasites has not been investigated in Kyrgyzstan, nor has the role of animals in the transmission of these ZDs been quantified. Therefore, burden assessment at this moment is not feasible.

Only sequelae that have a solid proven causal relationship with the pathogen have been included in the disease models we used. Reactive arthritis, irritable bowel syndrome and GBS are evidence based sequelae of campylobacteriosis [72]. However, we followed the conservative assumption of Kirk et al. that the relation between some sequelae were not sufficiently proven in middle and high-mortality countries [35]. Most of the burden of salmonellosis is due to YLLs, mainly deaths caused by iNTS. The sensitivity analysis (Table 3) illustrates the influence of the proportion of iNTS:NTS. This underlines the importance of investigating the incidence of iNTS in Kyrgyzstan and is in line with the findings of Ao et al. [46] conclude that there is a lack of population-based incidence data on iNTS.

In a limited-means setting such as Kyrgyzstan it is inevitable for policy makers to prioritize health care needs. The DALY provides one tool to do so, but is by itself not sufficient [73]. In the application of the DALY by healthcare legislators, it is important to look at the presented figures in a wider context. DALYs should be combined with for example, economic parameters in cost-utility analyses [74]. It is also important to realize that the DALY might not capture the full effect of the disease and that a disease might have bigger impact than just on the ones directly affected [75]. Especially ZDs often cause economic loss in livestock production as well [21,76]. Where in this paper we have only quantified the human burden, it makes sense to extend the work with the assessment of the economic impact of the disease in both humans and animals. Furthermore, it is advised to conduct an integrated approach in disease intervention and prevention where both veterinary and human health officials work together [3].

## Supporting Information

S1 Supporting Information Systematic review search construct. (DOCX)

S2 Supporting Information Prisma checklist. (DOCX)

S3 Supporting Information Data collection. (XLSX)

S4 Supporting Information DALY methodology. (DOCX)
