# Supplementary material for: The Burden of Zoonoses in Kyrgyzstan: A Systematic Review
Source: PLoS Negl Trop Dis. 2016 Jul 7;10(7):e0004831. doi: 10.1371/journal.pntd.0004831 (PMC4936671; doi:10.1371/journal.pntd.0004831)
Supplement: S1 Supporting Information — (DOCX) [file pntd.0004831.s001.docx]

# Supporting information 1 - Systematic review/search construct

**Table S1-1.** Databases and other sources searched.

| Database/source | Website |
| --- | --- |
| Pubmed | <http://www.ncbi.nlm.nih.gov/pubmed/> |
| OVID | <http://gateway.ovid.com/> |
| Web of science | <https://isiknowledge.com/> |
| Scopus | <http://www.scopus.com/> |
| Google Scholar | <https://scholar.google.com/> |
| WHO Global Health Library | <http://www.globalhealthlibrary.net/php/index.php> |
| World Organisation for Animal Health | <http://www.oie.int/doc/en_index.php> |
| eLibrary | <http://elibrary.ru> |
| Eastview | <http://www.eastview.com> |
| State Sanitary Epidemiological Department |  |
| Articles and abstracts of national scientific conferences |  |
| Government reports |  |

**Table S1-2.** Zoonotic diseases and additional keywords and pathogens name(s) used in the database searches.

| Disease | Additional keywords | Pathogen name | Complete search term |
| --- | --- | --- | --- |
| Parasitic Zoonoses | | | |
| Alveolar echinococcosis | Alveolar hydatidosis  Fox tapeworm | *Echinococcus multilocularis* | (Alveolar echinococcosis OR fox tapeworm OR Alveolar hydatidosis OR Echinococcus multilocularis) |
| Cystic echinococcosis | Hydatid disease  Hydatidosis  Hydatid cyst  Dog Tapeworm | *Echinococcus granulosus* | (Hydatid disease OR Hydatidosis OR Hydatid cyst OR Dog Tapeworm OR Echinococcus granulosus) |
| Toxoplasmosis | Toxoplasma | *Toxoplasma gondii* | (Toxoplasmosis OR Toxoplasma OR Toxoplasma gondii) |
| Viral Zoonoses | | | |
| Rabies |  | Rabies virus | (Rabies OR Rabies virus) |
| Bacterial Zoonoses | | | |
| Anthrax |  | *Bacillus anthracis* | (Anthrax OR Bacillus anthracis) |
| Brucellosis |  | *Brucella*  *B. abortus*  *B. melitensis* | (Brucellosis OR Bang's disease OR Crimean fever OR undulant fever OR Brucella melitensis OR Brucella abortus OR Brucella suis OR Brucella canis) |
| Salmonellosis | Non-typhoidal salmonella, Non-typhoidal salmonellosis, NTS | *Salmonella* | (Salmonellosis OR Salmonella OR Non-typhoidal salmonella OR NTS) |
| Campylobacteriosis |  | *Campylobacter* | (Campylobacteriosis OR Campylobacter) |

## Search for “Cystic echinococcosis” used as example:

Pubmed search: (Kyrgyzstan AND (Hydatid disease OR Hydatidosis OR Hydatid cyst OR Dog Tapeworm OR Echinococcus granulosus))

Formal notation:

("kyrgyzstan"[MeSH Terms] OR "kyrgyzstan"[All Fields]) AND (("echinococcosis"[MeSH Terms] OR "echinococcosis"[All Fields] OR ("hydatid"[All Fields] AND "disease"[All Fields]) OR "hydatid disease"[All Fields]) OR ("echinococcosis"[MeSH Terms] OR "echinococcosis"[All Fields] OR "hydatidosis"[All Fields]) OR ("echinococcosis"[MeSH Terms] OR "echinococcosis"[All Fields] OR ("hydatid"[All Fields] AND "cyst"[All Fields]) OR "hydatid cyst"[All Fields]) OR ("cestode infections"[MeSH Terms] OR ("cestode"[All Fields] AND "infections"[All Fields]) OR "cestode infections"[All Fields] OR ("dog"[All Fields] AND "tapeworm"[All Fields]) OR "dog tapeworm"[All Fields]) OR ("echinococcus granulosus"[MeSH Terms] OR ("echinococcus"[All Fields] AND "granulosus"[All Fields]) OR "echinococcus granulosus"[All Fields]))

OVID:

1 Hydatid disease OR Hydatidosis OR Hydatid cyst OR Dog Tapeworm

OR Echinococcus granulosus [Including Limited Related Terms]

2 Kyrgyzstan [Including Limited Related Terms]

3 1 AND 2

Web of Science:

TS=Kyrgyzstan AND TS=(Hydatid disease OR Hydatidosis OR Hydatid cyst OR Dog Tapeworm OR Echinococcus granulosus)

TS=(Kyrgyzstan OR Kirghizia OR Kirgizstan OR Kyrgyz Republic) AND TS=(Hydatid disease OR Hydatidosis OR Hydatid cyst OR Dog Tapeworm OR Echinococcus granulosus)

Scopus:

( TITLE-ABS-KEY ( hydatid disease OR hydatidosis OR hydatid cyst OR dog tapeworm OR echinococcus granulosus ) ) AND ( TITLE-ABS-KEY ( kyrgyzstan ) )

( TITLE-ABS-KEY ( echinococcus granulosus ) ) AND ( TITLE-ABS-KEY ( kyrgyzstan ) )
