# Supplementary material for: The Burden of Zoonoses in Kyrgyzstan: A Systematic Review
Source: PLoS Negl Trop Dis. 2016 Jul 7;10(7):e0004831. doi: 10.1371/journal.pntd.0004831 (PMC4936671; doi:10.1371/journal.pntd.0004831)
Supplement: S4 Supporting Information — (DOCX) [file pntd.0004831.s004.docx]

# Supporting information S4 - DALY methodology

## Cystic echinococcosis (CE)

The burden calculation for CE is based on Torgerson et al. [[1](#_ENREF_1)] and disease incidence based on [[2-4](#_ENREF_2)]. Figure S4-1A shows the different outcomes departing from infection with CE for the group of patients seeking treatment and the group not seeking treatment, Figure S4-1B shows the different elements used in the DALY calculation. Table S4-1 summarizes the applied DALY parameters.

**Figure S4-1A.** Disease model for CE. CNS CE = Cystic echinococcosis of the Central Nervous System.

**Figure S4-1B.** DALY formula for CE

**Table S4-1.** DALY parameters for CE.

| **Parameter** | **Distribution** | **Value (95% Range)** | **Reference** |
| --- | --- | --- | --- |
| Disability weight pulmonary CE treated | Fixed | 0.123 | [[1](#_ENREF_1)] |
| Disability weight hepatic CE treated | Fixed | 0.192 | [[1](#_ENREF_1)] |
| Disability weight CNS CE treated | Fixed | 0.221 | [[1](#_ENREF_1)] |
| Disability weight pulmonary CE untreated | Fixed | 0.015 | [[1](#_ENREF_1)] |
| Disability weight hepatic CE untreated | Fixed | 0.012 | [[1](#_ENREF_1)] |
| Disability weight CNS CE untreated | Fixed | 0.054 | [[1](#_ENREF_1)] |
| Proportions abdominal pelvic | Uniform(min=0.6,max=0.75) | 0.68 [0.60-0.75] | [[5-7](#_ENREF_5)] |
| Proportion respiratory disease | Uniform(min=0.15,max=0.30) | 0.23 [0.15-0.30] | [[5-7](#_ENREF_5)] |
| Proportion CNS disease | Uniform(min=0.005, max=0.03) | 0.018 [0.006-0.03] | [[7](#_ENREF_7)] |
| Duration disease untreated (years) | *Lifelong* |  | [[1](#_ENREF_1)] |
| Duration disease treated (years) | Fixed | 2 | [[1](#_ENREF_1)] |
| Age of onset | Dirichlet(8,30,97,73,41,29,13)  for age groups(<10,10-20,20-30,30-40,40-50,50-60,>60) | 33 (5-65) | [[3](#_ENREF_3)] |
| Incidence treated | Uniform(min=901, max=1,009) | 955 (904-1,006) | See text  [[2-4](#_ENREF_2)] |
| Incidence untreated | Pert(min=901, mode=1,009, max=4,415) | 1,435 (929-2,835) | See text  [[2-4](#_ENREF_2)] |

## Alveolar echinococcosis (AE)

The burden calculation for AE is based on Torgerson et al. [[1](#_ENREF_1),[8](#_ENREF_8),[9](#_ENREF_9)], the disease incidence is based on [[2-4](#_ENREF_2)]. Figure S4-2A shows the different outcomes departing from infection with AE, Figure S4-2B shows the different elements used in the DALY calculation. Table S4-2 summarizes the applied DALY parameters.

**Figure S4-2A.** Disease model for AE.

**Figure S4-2B.** DALY formula for AE

**Table S4-2.** Parameters used in DALY calculation of AE.

| **Parameter** | **Distribution** | **Value (95% Range)** | **Reference** |
| --- | --- | --- | --- |
| Disability weight disease | Fixed | 0.123 | [[10](#_ENREF_10)] |
| Duration disease (years) | Uniform(8,11) | 7.5 (6.1-8.9) | [[8](#_ENREF_8),[9](#_ENREF_9)] |
| Age of onset | Dirichlet(8,30,97,73,41,29,13)  for age groups(<10,10-20,20-30,30-40,40-50,50-60,>60) | 33 (5-65) | [[3](#_ENREF_3)] |
| Incidence | PERT(min=148, mode=166, max=725) | 236 (153-466) | See text  [[2-4](#_ENREF_2)] |
|  |  |  |  |

### Underestimation in echinococcosis

Prevalence data for AE was available from Bebezov et al. (*unpublished manuscript)*, who performed in 2012 an ultrasound screening of 1,617 subjects in the Alay Valley in the Osh Oblast of which 106 had lesions suggestive of AE, which is a prevalence of 6.6%. 28 of these subjects underwent surgery, in which 26 were confirmed by histology and or PCR as AE. Within the Osh Oblast the Alay district has a high incidence of cases: 58/100,000/year [[2](#_ENREF_2)]. The estimated true incidence of AE was calculated by assuming that the disease can be modelled as a stable disease. The delay in onset of symptoms after infection can be up to 15 years; the duration of the clinical disease is assumed to be 10 years [[11](#_ENREF_11)]. Assuming an average infection period of 25 years (latent period + disease duration) and assuming all ultrasonic cases become surgical cases, we would expect 282.64/100,000 cases in the region per year, or 4.9 times more cases than the reported cases. This factor was used to calculate a maximum for the cases, for which the distribution was modelled as a PERT distribution with a minimum of the reported cases, a mode of 12% higher, based on the underreporting observed by Raimkylov et al. [[2](#_ENREF_2)] and the mentioned maximum. For CE we assume that the group seeking treatment is represented by the uniform range between the official incidence and that incidence corrected for 12% underreporting. The group not seeking treatment is at least as big as the incidence of cases seeking treatment based on [[1](#_ENREF_1)] and modelled as pert distribution with a maximum of 4.9 times the minimum and a mode corrected for 12% underreporting..

## Rabies

The burden calculation for rabies is based on [[12-14](#_ENREF_12)], the disease incidence is based on [[14](#_ENREF_14),[15](#_ENREF_15)]. Figure S4-3A shows the outcome departing from infection with rabies, Figure S4-3B shows the different elements used in the DALY calculation. We assume a model in which rabies only causes death and therefore there are no YLDs contributed by the disease. Table S4-3 summarizes the applied DALY parameters.

**Figure S4-3A.** Incidence based disease model for rabies.

**Figure S4-3B.** DALY formula for rabies.

**Table S4-3.** Parameters used in DALY calculation for rabies.

| **Parameter** | **Distribution** | **Value (95% Range)** | **Reference** |
| --- | --- | --- | --- |
| RP^1^ | Fixed | 0.474 | [[12](#_ENREF_12)] |
| PP^2^ | Fixed | 0.99 | [[12](#_ENREF_12)] |
| DP^3^ | Fixed | 0.19 | [[13](#_ENREF_13)] |
| Age of onset | Gamma(shape=1.54, rate=0.088) | 17.5 (1.3-54.0) | [[13](#_ENREF_13)] |
| Incidence of dog bites | Fixed | 0.01996 | [[16](#_ENREF_16)] See text |
| Population | Fixed | 5,663,133 | Kyrgyz stat |
| Incidence | Uniform(min=1, max=10) | 5 (1-10) | [[14](#_ENREF_14),[15](#_ENREF_15)], See text |
| ^1^ the probability that the bite is from a rabid animal  ^2^ the probability of receiving PEP  ^3^ the probability that in the absence of PEP the bite victim develops rabies | | | |

### Underestimation in rabies

We assumed that the incidence of rabies in Kyrgyzstan in 2013 is distributed between the official reported incidence as lower limit and the maximum incidence based on the methodology of Hampson [[14](#_ENREF_14)] as upper limit. The maximum number of yearly rabies cases (Max_cases) is calculated using the product of the population, the bite incidence, the probability that the bite is from a rabid animal (RP), one minus the probability of receiving PEP (1-PP) and the probability that in the absence of PEP the bite victim develops rabies (DP). This is displayed in Formula S4-1. In the first 9 months of 2015 10,350 animal bites were reported by the Department of Disease Prevention and Sanitary Inspection, of which 84% were dog bites. This results in a yearly incidence of dog bites of 11,542, or 0.01996 dog bites/100,000 persons in 2015 (data is available from [[16](#_ENREF_16)]). We assume the same incidence for 2013.

| Max_cases = population * (bite incidence * RP) * (1-PP) * DP | (S4-1) |
| --- | --- |
|  | |

## Brucellosis

The burden calculation for brucellosis is based on Kirk et al. [[17](#_ENREF_17)], the disease incidence is based on [[18-20](#_ENREF_18)]. Figure S4-4A shows the different outcomes departing from infection with *Brucella*, Figure S4-4B shows the different elements used in the DALY calculation. Table S4-4 summarizes the applied DALY parameters.

**Figure S4-4A.** Incidence based disease model for brucellosis.

**Figure S4-4B.** DALY formula for brucellosis.

**Table S4-4.** Parameters used in DALY calculation for brucellosis.

| **Parameter** | **Distribution** | **Value (95% Range)** | **Reference** |
| --- | --- | --- | --- |
| Proportion acute -severe | Fixed | 0.3 | [[17](#_ENREF_17)] |
| Proportion acute -moderate | Fixed | 0.3 | [[17](#_ENREF_17)] |
| Proportion chronic | Fixed | 0.4 | [[17](#_ENREF_17)] |
| Proportion orchitis (males) | Fixed | 0.055 | [[17](#_ENREF_17)] |
| Disability weight acute severe | Fixed | 0.210 | [[17](#_ENREF_17)] |
| Disability weight acute moderate | Fixed | 0.053 | [[17](#_ENREF_17)] |
| Disability weight chronic | Fixed | 0.079 | [[17](#_ENREF_17)] |
| Disability weight orchitis | Fixed | 0.097 | [[17](#_ENREF_17)] |
| Duration chronic (years) | PERT(min=0.25, max=2, mode=0.5) | 0.66 y (0.30-1.35) | [[17](#_ENREF_17)] |
| Duration acute (years) | PERT(min=7/365, max=21/365, mode=14/365) | 14 d (9-19) | [[17](#_ENREF_17)] |
| Proportion fatal | Fixed | 0.005 | [[17](#_ENREF_17)] |
| Age of onset | Proportion(0.03,0.29,0.24,0.16, 0.13,0.12,0.03)  for age groups(0-15,15-25,25-35,35-45,45-55,55-70) |  | [[17](#_ENREF_17)] |
| Incidence | Pert(min=1,364, max=6,274, mode=1,949) | 2,427 (1,468-4,430) | See Text [[18-20](#_ENREF_18)] |
|  | | | |

### Underestimation in brucellosis

Prevalence data was collected by Bonfoh et al. and was used to address the underestimation in the incidence of brucellosis [[19](#_ENREF_19)]. Based on the assumptions that the average duration of seropositivity is 10.9 years and 10% of seroconversions lead to clinical disease [[21](#_ENREF_21)], the incidence of disease can be estimated from prevalence, using formula S4-2 [[22](#_ENREF_22)].

| P / (1-P) = I * D | (S4-2) |
| --- | --- |

Bonfoh estimated the seroprevalence of brucellosis in Kyrgyzstan in 2006 based on 1,774 human sera using different methods [[19](#_ENREF_19)]. The results of the Rose Bengal test, ELISA and the Huddleson test were compared by Dürr et al. using Bayesian methods to calculate an estimate of the true seroprevalence [[20](#_ENREF_20)]. Based on this true prevalence (7%) and formula S4-2, an annual incidence of 690/100.000 cases/year is calculated; of these 690 cases 10% leads to clinical disease: 69/100.000, which is close to the 74.4/100.00 cases/year reported for 2006 by the Department of State Sanitary and Epidemiological Supervision of the Ministry of Health of the Kyrgyz Republic. However, it is argued that the 10% of seroconversions resulting in clinical cases is a conservative estimate. If we assume this to be 50%, as reported by Bonfoh et al. [[19](#_ENREF_19)], the underestimation factor is 4.6. Another assumption is that only severe acute or chronic cases seek medical assistance, therefore the reported cases represent 70% of the total cases. We modelled the uncertainty around the incidence as PERT distribution with the officially reported yearly incidence as minimum, the mode as the cases corrected for percentage seeking medical assistance, and as the maximum the officially reported cases multiplied by the level of underestimation (4.6).

## Campylobacter

The burden calculation for campylobacteriosis is based on Kirk et al. [[17](#_ENREF_17)], the disease incidence is based on [[18](#_ENREF_18),[23-26](#_ENREF_23)]. Figure S4-5A shows the different outcomes departing from infection with *Campylobacter*, Figure S4-5B shows the different elements used in the DALY calculation. The outcome for gastroenteritis (GE) below the age of five (GE<5) is calculated separately from the same outcome over five years of age (GE>5). Table S4-5 summarizes the applied DALY parameters.

**Figure S4-5A.** Incidence based disease model for campylobacteriosis. Gastroenteritis (GE) can be followed by the sequela Guillain-Barré Syndrome (GBS).

**Figure S4-5B.** DALY formula for campylobacteriosis.

**Table S4-5.** Parameters used in DALY calculation for campylobacteriosis.

| **Parameter** | **Distribution** | **Value (95% Range)** | **Reference** |
| --- | --- | --- | --- |
| Proportion GE severe | Fixed | 0.02 | [[17](#_ENREF_17)] |
| Proportion GE moderate | Fixed | 0.25 | [[17](#_ENREF_17)] |
| Proportion GE mild | Fixed | 0.73 | [[17](#_ENREF_17)] |
| Disability weight GE severe | Fixed | 0.281 | [[17](#_ENREF_17)] |
| Disability weight GE moderate | Fixed | 0.202 | [[17](#_ENREF_17)] |
| Disability weight GE mild | Fixed | 0.061 | [[17](#_ENREF_17)] |
| Disability weight GBS | Fixed | 0.445 | [[17](#_ENREF_17)] |
| Duration GE <5y | PERT(min=4.3, max=8.4, mode=6.4)(days) | 6.4 (4.9-7.8) days | [[17](#_ENREF_17)] |
| Duration GE >5y | Fixed | 2.8 days | [[17](#_ENREF_17)] |
| Duration GBS | Fixed | Lifelong | [[17](#_ENREF_17)] |
| Age of onset GE <5y | Fixed | 2.5y | See text |
| Age of onset GE >5y | Proportion(0.26,0.53,0.21)  For age groups(5-14,15-54,>55) |  | See text |
| Age of onset GBS | Idem age of onset GE |  | [[17](#_ENREF_17)] |
| Incidence GE <5y | Uniform(min=477, max=70,877) | 39,929 (10,507 -69,074) | [[18](#_ENREF_18),[23](#_ENREF_23),[24](#_ENREF_24)] See text |
| Fatality GE <5y | Uniform(min=10, max=13) | 12 (10-13) | [[24](#_ENREF_24),[27](#_ENREF_27)] See text |
| Incidence GE >5y | Uniform(min=509, max=75,707) | 42,569 (11,224 - 73,707) | [[18](#_ENREF_18),[25](#_ENREF_25),[26](#_ENREF_26)] See text |
| Fatality GE >5y | Uniform(min=9, max= 13) | 11 (9-13) | [[18](#_ENREF_18)] See text |
| Incidence proportion GBS | Uniform(min=0.0002,max=0.0009) | 0.00055 (0.00022 - 0.00089) | [[28](#_ENREF_28)] |
| Proportion fatal GBS | PERT(min=0.024, max=0.06, mode=0.041) | 0.041 (0.029-0.054) | [[17](#_ENREF_17)] |
|  | | | |

### Underestimation in campylobacteriosis

The incidence of GE cases caused by *Campylobacter* in 2013 in Kyrgyzstan was estimated to be following a uniform distribution with a minimum of the product of the etiological fraction reported in the reviews of Lanata et al. and Walker et al. due to campylobacteriosis [[24](#_ENREF_24),[26](#_ENREF_26)] and the cases of diarrhoea reported by the Government Sanito-Epidemiology Unit (29,045 reported cases of acute intestinal infections) [[18](#_ENREF_18)]. The maximum for this distribution was calculated by multiplying the etiological fraction by the estimated incidence of diarrhoea as reported in the reviews of Walker et al. [[23](#_ENREF_23),[25](#_ENREF_25)] in the European region. Since the duration of the disease, the incidence and the etiological fraction between the under-5-years and over-5-years group differs, disease burden was calculated separately for the two age groups. The methodology of using the etiological fraction and addressing incidence separately in the two age groups was derived from the methodology of the WHO Foodborne Disease Burden Epidemiology Reference Group study in which the global burden of food borne pathogens was estimated [[17](#_ENREF_17)].

Mortality due to GE for the age group under-5-years was calculated using the WHO Child Health Epidemiology Reference Group mortality incidence due to diarrhoea for Kyrgyzstan in 2013, multiplied by the etiological fraction as described above [[29](#_ENREF_29)]. Mortality for the age group over-5-years was calculated using the same methodology but based on the country specific GBD 2010 diarrhoeal mortality estimates for the different age groups. The uncertainty range from the GBD 2010 estimate of diarrheal deaths was applied to model uncertainty around the estimates (81.7% to 114.6% around the point estimate) [[30](#_ENREF_30)].

The proportion per age group was derived from the diarrhoeal incidence multiplied by the etiological fraction per age group; the proportion was the relative incidence of diarrhoea caused by the specific pathogen.

## Non-typhoidal salmonellosis

The burden calculation for non-typhoidal salmonellosis (NTS) is based on Kirk et al. [[17](#_ENREF_17)], the disease incidence is based on [[18](#_ENREF_18),[23-26](#_ENREF_23)]. Figure S4-5A shows the different outcomes departing from infection with non-typhoidal *Salmonella* (NTS) Figure S4-5B shows the different elements used in the DALY calculation. The outcome for gastroenteritis (GE) below the age of five (GE<5) is calculated separately from the same outcome over five years of age (GE>5). Table S4-5 summarizes the applied DALY parameters.

**Figure S4-6A.** Incidence based disease model for non-typhoidal *Salmonella* (NTS) that causes acute gastroenteritis (GE) and the invasive form of the disease (iNTS).

**Figure S4-6B.** DALY formula for NTS.

**Table S4-6.** Parameters used in DALY calculation for non-typhoidal salmonellosis.

| **Parameter** | **Distribution** | **Value (95% Range)** | **Reference** |
| --- | --- | --- | --- |
| Proportion GE severe | Fixed | 0.02 | [[17](#_ENREF_17)] |
| Proportion GE moderate | Fixed | 0.25 | [[17](#_ENREF_17)] |
| Proportion GE mild | Fixed | 0.73 | [[17](#_ENREF_17)] |
| Disability weight GE severe | Fixed | 0.281 | [[17](#_ENREF_17)] |
| Disability weight GE moderate | Fixed | 0.202 | [[17](#_ENREF_17)] |
| Disability weight GE mild | Fixed | 0.061 | [[17](#_ENREF_17)] |
| Disability weight iNTS | Fixed | 0.281 | [[17](#_ENREF_17)] |
| Duration GE <5 y | Min=4.3, max=8.4, mode=6.4 (days) | 6.4d (4.9-7.8) | [[17](#_ENREF_17)] |
| Duration GE >5 y | Fixed | 2.8 d | [[17](#_ENREF_17)] |
| Duration iNTS | PERT(min=7d, max=56d, mode=28d) | 29 d (13-47) | [[17](#_ENREF_17)] |
| Incidence GE <5y | Uniform(min=11595, max=87089) | 49,345 (13,482-85,203) | [[18](#_ENREF_18),[23](#_ENREF_23),[24](#_ENREF_24)] See text |
| Incidence GE >5y | Uniform(min=4276, max=32118) | 18,202 (4,971-31,422) | [[18](#_ENREF_18),[25](#_ENREF_25),[26](#_ENREF_26)] See text |
| Incidence proportion iNTS | 1/28 | 0.0357 | [[31](#_ENREF_31)] See text |
| Fatality GE <5y | PERT(min=12,max=17, mode=14) | 14 (13-16) | [[24](#_ENREF_24),[27](#_ENREF_27)] See text |
| Mortality GE >5 y | PERT(min=4, max=6, mode=5) | 5 (4-6) | [ref] See text |
| Proportion fatal iNTS | PERT(min=0.05, max=0.20, mode=0.1) | 0.11 (0.06-0.17) | [[17](#_ENREF_17)] |
| Age of onset GE <5y | Fixed | 2.5 y | See text |
| Age of onset GE >5y | Proportion(0.26,0.53,0.21)  for age groups(5-14,15-54,>54) |  | See text |
| Age of onset iNTS | Proportion(0.22,0.1,0.43,0.25)  For age groups(<5,5-14,15-24,25-64,>65) |  | [[31](#_ENREF_31)] |
|  | | | |

### Underestimation in non-typhoidal salmonellosis

The incidence and the mortality of GE caused by non-typhoidal salmonellosis (NTS) in 2013 was estimated following the same approach used for campylobacteriosis described above. The incidence of invasive non-typhoidal salmonellosis (iNTS) is calculated by applying a ratio of 1:28 to the incidence of NTS cases (iNTS:NTS) based on Ao et al. [[31](#_ENREF_31)]. However, a large spread in relative incidence of iNTS is described by this author. Mortality for iNTS was defined as 10% (5-20%) of the cases of iNTS [[17](#_ENREF_17)].

## Toxoplasmosis

The burden calculation for congenital toxoplasmosis is based on [[32-34](#_ENREF_32)], the disease incidence is based on [[18-20](#_ENREF_18)]. Figure S4-4A shows the different outcomes departing from infection with toxoplasmosis during pregnancy, Figure S4-4B shows the different elements used in the DALY calculation. Table S4-4 summarizes the applied DALY parameters.

**Figure S4-7A.** Disease outcome model for congenital toxoplasmosis.

**Figure S4-7B.** DALY formula for congenital toxoplasmosis.

**Table S4-7.** Parameters used in DALY calculation for congenital toxoplasmosis.

| **Parameter** | **Distribution** | **Value (95% Range)** | **Reference** |
| --- | --- | --- | --- |
| Proportion chorioretinitis at birth | Beta(142, 908) | 0.13 (0.11–0.16) | [[32](#_ENREF_32)] |
| Proportion chorioretinitis later in life | Uniform(0.086, 0.237) | 0.16 (0.09–0.23) | [[33](#_ENREF_33)] |
| Proportion hydrocephalus | Beta(17, 841) | 0.02 (0.01–0.03) | [[32](#_ENREF_32)] |
| Proportion neonatal death | Triangle(min=0.01,max=0.09, mode=0.03) | 0.04 (0.02-0.08) | [[34](#_ENREF_34)] |
| Proportion foetal death | Beta(10,1203) | 0.008 (0.004-0.014) | [[32](#_ENREF_32)] |
| Proportion intracranial calcifications | Beta(89,750) | 0.106 (0.086-0.128) | [[32](#_ENREF_32)] |
| Neonates with CNS abnormalities | Beta(4,103) | 0.035 (0.010-0.081) | [[32](#_ENREF_32)] |
| Disability weight hydrocephalus & CNS abnormalities | Fixed | 0.36 | [[32](#_ENREF_32)] |
| Disability weight chorioretinitis | Fixed | 0.033 | [[33](#_ENREF_33)] |
| Disability weight intracranial calcifications | Fixed | 0.01 | [[32](#_ENREF_32)] |
| Duration outcomes except chorioretinitis later in life | Fixed | Lifelong, onset at birth | [[32](#_ENREF_32)] |
| Duration chorioretinitis later in life | Fixed | Lifelong, onset at age 10 | [[32](#_ENREF_32)] |
| Number of cases 2013 | Lognormal(mean= log(216) ,sdlog=(log(261)-log(216))/1.96) | 216 (179-261) | [[35](#_ENREF_35)] See text |

### Underestimation in toxoplasmosis

Minbaeva et al. estimated, based on seroprevalence per age group, that in 2008 173 (136-216) babies were born from mothers who seroconverted during pregnancy out of 127,322 live births in Kyrgyzstan [[35](#_ENREF_35)]. Assuming the stability of prevalence over time and correcting for an increase in live births we would assumed 216 (179-261) children born with congenital toxoplasmosis in 2013 from seroconverted mothers. Since the incidence data is directly derived from a prevalence study, no correction for underestimation was made.

## Origin of the data

Figure S4-8 displays a diagram of the different variables used in the DALY calculation per disease and their origin, the numbers within the brackets are the references to the original data.

**Figure S4-8.** Summary of the different variables used in the DALY calculation per disease and their origin.

# References

1. Torgerson PR, Devleesschauwer B, Praet N, Speybroeck N, Willingham AL, et al. (2015) World Health Organization Estimates of the Global and Regional Disease Burden of 11 Foodborne Parasitic Diseases, 2010: A Data Synthesis. PLoS Med 12: e1001920.

2. Raimkylov KM, Kuttubaev OT, Toigombaeva VS (2015) Epidemiological analysis of the distribution of cystic and alveolar echinococcosis in Osh Oblast in the Kyrgyz Republic, 2000-2013. J Helminthol 89: 651-654.

3. Usubalieva J, Minbaeva G, Ziadinov I, Deplazes P, Torgerson PR (2013) Human alveolar echinococcosis in Kyrgyzstan. Emerg Infect Dis 19: 1095-1097.

4. Carabin H, Balsera-Rodríguez FJ, Rebollar-Sáenz J, Benner CT, Benito A, et al. (2014) Cystic Echinococcosis in the Province of Álava, North Spain: The Monetary Burden of a Disease No Longer under Surveillance. PLoS Negl Trop Dis 8: e3069.

5. Budke CM, Deplazes P, Torgerson PR (2006) Global Socioeconomic Impact of Cystic Echinococcosis. Emerging Infectious Diseases 12: 296-303.

6. Eckert J, Gemmell M, Meslin F, Pawlowski Z (2001) WHO/OIE Manual on echinococcosis in humans and animals: a public health problem of global concern. 2001. Disponible en: whqlibdoc who int/publications/2001 X 929044522.

7. Turgut M (2002) Hydatidosis of central nervous system and its coverings in the pediatric and adolescent age groups in Turkey during the last century: a critical review of 137 cases. Child's Nervous System 18: 670-683.

8. Torgerson PR, Keller K, Magnotta M, Ragland N (2010) The Global Burden of Alveolar Echinococcosis. PLoS Negl Trop Dis 4: e722.

9. Torgerson PR, Schweiger A, Deplazes P, Pohar M, Reichen J, et al. (2008) Alveolar echinococcosis: from a deadly disease to a well-controlled infection. Relative survival and economic analysis in Switzerland over the last 35 years. J Hepatol 49: 72-77.

10. Devleesschauwer B, Haagsma JA, Angulo FJ, Bellinger DC, Cole D, et al. (2015) Methodological Framework for World Health Organization Estimates of the Global Burden of Foodborne Disease. PLoS ONE 10: e0142498.

11. Eckert J, Deplazes P (2004) Biological, epidemiological, and clinical aspects of echinococcosis, a zoonosis of increasing concern. Clinical microbiology reviews 17: 107-135.

12. Al-Shamahy HA, Wright SG (2001) A study of 235 cases of human brucellosis in Sana'a, Republic of Yemen. East Mediterr Health J 7: 238-246.

13. Shim E, Hampson K, Cleaveland S, Galvani AP (2009) Evaluating the cost-effectiveness of rabies post-exposure prophylaxis: A case study in Tanzania. Vaccine 27: 7167-7172.

14. Hampson K, Coudeville L, Lembo T, Sambo M, Kieffer A, et al. (2015) Estimating the global burden of endemic canine rabies. PLoS Negl Trop Dis 9: e0003709.

15. Department of State Sanitary and Epidemiological Supervision of the Ministry of Health of the Kyrgyz Republic (2013) Overview of infectious diseases for the months of January-December 2013.

16. AKIpress (2015) 10,305 people in Kyrgyzstan apply to hospital with animal bites since start of 2015. Bishkek.

17. Kirk MD, Pires SM, Black RE, Caipo M, Crump JA, et al. (2015) World Health Organization Estimates of the Global and Regional Disease Burden of 22 Foodborne Bacterial, Protozoal, and Viral Diseases, 2010: A Data Synthesis. PLoS Med 12: e1001921.

18. National Statistical Committee of the Kyrgyz Republic (2015) Healthcare official statistics - Number of new cases of infectious and parasitic diseases.

19. Bonfoh B, Kasymbekov J, Duerr S, Toktobaev N, Doherr MG, et al. (2012) Representative Seroprevalences of Brucellosis in Humans and Livestock in Kyrgyzstan. Ecohealth 9: 132-138.

20. Durr S, Bonfoh B, Schelling E, Kasymbekov J, Doherr MG, et al. (2013) Bayesian estimation of the seroprevalence of brucellosis in humans and livestock in Kyrgyzstan. Rev Sci Tech 32: 801-815.

21. Dean AS, Crump L, Greter H, Schelling E, Zinsstag J (2012) Global burden of human brucellosis: a systematic review of disease frequency.

22. Dohoo IR, Martin W, Stryhn HE (2007) Veterinary epidemiologic research. Prince Eward Island: AVC Inc.

23. Walker CLF, Rudan I, Liu L, Nair H, Theodoratou E, et al. (2013) Global burden of childhood pneumonia and diarrhoea. The Lancet 381: 1405-1416.

24. Lanata CF, Fischer-Walker CL, Olascoaga AC, Torres CX, Aryee MJ, et al. (2013) Global causes of diarrheal disease mortality in children< 5 years of age: a systematic review. Plos one 8: e72788.

25. Walker CF, Black R (2010) Diarrhoea morbidity and mortality in older children, adolescents, and adults. Epidemiology and infection 138: 1215-1226.

26. Walker CF, Sack D, Black RE (2010) Etiology of diarrhea in older children, adolescents and adults: a systematic review. PLoS Negl Trop Dis 4: e768-e768.

27. Liu L, Johnson HL, Cousens S, Perin J, Scott S, et al. (2012) Global, regional, and national causes of child mortality: an updated systematic analysis for 2010 with time trends since 2000. The Lancet 379: 2151-2161.

28. Mangen M-JJ, Plass D, Havelaar AH, Gibbons CL, Cassini A, et al. (2013) The Pathogen- and Incidence-Based DALY Approach: An Appropriated Methodology for Estimating the Burden of Infectious Diseases. PLoS ONE 8: e79740.

29. Child Health Epidemiology Reference Group (2011) CHERG Child Causes of Death Annual Estimates by Country, 2000-2010.

30. Murray CJ, Ezzati M, Flaxman AD, Lim S, Lozano R, et al. (2013) GBD 2010: design, definitions, and metrics. The Lancet 380: 2063-2066.

31. Ao TT (2013) Global Burden of Invasive Nontyphoidal Salmonella Disease, 2010. On the Cover 2012: 941.

32. Havelaar A, Kemmeren J, Kortbeek L (2007) Disease burden of congenital toxoplasmosis. Clinical Infectious Diseases 44: 1467-1474.

33. Torgerson PR, Mastroiacovo P (2013) The global burden of congenital toxoplasmosis: a systematic review. Bulletin of the World Health Organization 91: 501-508.

34. Kortbeek LM, Hofhuis A, Nijhuis CD, Havelaar AH (2009) Congenital toxoplasmosis and DALYs in the Netherlands. Mem Inst Oswaldo Cruz 104: 370-373.

35. Minbaeva G, Schweiger A, Bodosheva A, Kuttubaev O, Hehl AB, et al. (2013) Toxoplasma gondii infection in Kyrgyzstan: seroprevalence, risk factor analysis, and estimate of congenital and AIDS-related toxoplasmosis. PLoS neglected tropical diseases 7: e2043.
